# 

**Published:** 1864-12

**Authors:** 


### CHICAGO MEDICAL JOURNAL.

**Terms, $2.00 per Annum.**

### CONTENTS OF THE DECEMBER NUMBER.

PAGE.

ORIGINAL COMMUNICATIONS.

Report on Orthopedic Surgery, made at the Annual Meeting of the Illinois State Medical Society, convened in Chicago, May 3d, 1864. By David Prince, M. D., Jacksonville, Ill. .... 527

Communicated. .... 534

The Duties and Responsibilities of the Physician. An Essay read before the Stark Co. Med. Soc., Oct. 1st, 1864. By J. B. Hoag, M. D. 539

Clinical Lectures on Diseases of the Eye. By E. L. Holmes, M. D., of Chicago, Lecturer, on Diseases of the Eye and Ear in Rush Medical College—Artificial Pupil. .... 545

SELECTED.

Constitutional Syphilis. A Clinical Lecture, by Jonathan Hutchinson, F. R. C. S., Surgeon to the London Hospital .... 549

The Physiological Effects of Tobacco. By W. B. Richardson, M. A., M. D. .... 556

Report of a Case of Opisthotonos Cured by Ice. By Edward Howard, M. R. C. P. L. .... 557

Effects of Ergot of Wheat .... 558

Recovery in two cases of Acute Traumatic Tetanus. By O. Foster, Esq., M. R. C. S. .... 559

Editorial and Miscellaneous .... 562

Book Notices and Reviews .... 565

